# Knowledge, Attitudes, and Reported Practices Regarding Prediabetes Among Hypertensive Patients With Abdominal Obesity: A Cross‐Sectional Structural Equation Modeling Analysis

**DOI:** 10.1155/jdr/3315859

**Published:** 2026-10-10

**Authors:** Jian Chen, Xia Cao, Jinyun Zhang, Liting Zhou, Bofan Huang

**Affiliations:** ^1^ Affiliated Maternity and Child Health Care Hospital of Nantong University Nantong China ahnmc.com; ^2^ Hongqiao Subdistrict Community Health Service Center of Chongchuan District Nantong China

**Keywords:** abdominal obesity, attitudes, hypertension, knowledge, practices, prediabetes, structural equation modeling

## Abstract

**Background:**

We assessed prediabetes‐related knowledge, attitudes, and reported practices among adults with hypertension and abdominal obesity and examined whether their associations were compatible with a hypothesized KAP structure.

**Methods:**

This cross‐sectional questionnaire survey included 446 adults recruited from April to June 2025. A revision‐stage coding audit corrected P5 scoring. Because P4 and P6 had near‐zero, nonsignificant loadings, the primary practice construct comprised P1, P2, P3, and P5; full‐item models were retained as sensitivity analyses. Spearman correlations and structural equation modeling (SEM) were used.

**Results:**

Mean knowledge, attitude, and four‐item practice scores were 8.90 ± 2.14, 37.31 ± 3.68, and 10.75 ± 2.38. Correlations were 0.160 for knowledge–attitude, 0.173 for knowledge–practice, and 0.146 for attitude–practice (all *p* ≤ 0.002). The primary SEM showed acceptable fit (CMIN/DF = 2.127, RMSEA = 0.050, CFI = 0.925). Knowledge was associated with attitude (*β* = 0.160, *p* = 0.012), and attitude with practice (*β* = 0.281, *p* = 0.004); the direct knowledge–practice path was nonsignificant (*β* = 0.069, *p* = 0.402). The model‐based indirect association was 0.045 (95% CI 0.016–0.091; *p* = 0.004). Practice showed weak convergent reliability (AVE = 0.199; CR = 0.464), and attitude responses had marked ceiling effects.

**Conclusions:**

The findings suggest modest cross‐sectional associations among KAP domains and are compatible with the hypothesized structure but do not establish temporal ordering or mediation. Longitudinal studies using validated instruments and objective glycemic assessment are needed.

## 1. Introduction

Prediabetes is an intermediate glycemic state in which glucose concentrations are above the normal range but below diagnostic thresholds for diabetes. It is clinically important because it is associated with subsequent Type 2 diabetes mellitus (T2DM), cardiovascular disease, and other adverse outcomes [1, 2]. In China, national surveys have documented a substantial burden of diabetes and prediabetes, emphasizing the scale of the population potentially requiring earlier risk recognition and prevention [3, 4]. Randomized prevention trials have further shown that intensive lifestyle modification can delay or prevent progression to T2DM among people with impaired glucose regulation [5, 6]. These findings support prevention efforts before overt diabetes develops while also underscoring that cardiometabolic risk factors alone should not be equated with a laboratory diagnosis of prediabetes.

Hypertension and abdominal obesity are particularly relevant in this context because they commonly coexist with insulin resistance and other components of cardiometabolic dysfunction [7, 8]. Prediabetes itself is associated with increased cardiovascular risk [9], and hypertension and dysglycemia share several metabolic pathways and frequently occur together [10]. Current reviews therefore emphasize risk‐factor identification, weight management, physical activity, and dietary change as central components of prediabetes management [11]. Adults who have both hypertension and abdominal obesity may consequently represent a clinically important elevated‐risk group in whom recognition of prediabetes and understanding of preventive behaviors deserve attention, even when glycemic status has not yet been established objectively.

Knowledge–attitude–practice (KAP) surveys provide one approach to describing such prevention‐related gaps. They can characterize what people know about a condition, how they perceive its importance and preventability, and which relevant behaviors they report [12]. At the same time, KAP findings require cautious interpretation because reported knowledge, attitudes, and behaviors may not align, and cross‐sectional surveys cannot determine whether one domain precedes or causes another [13, 14]. A systematic review of prediabetes‐related KAP found persistent knowledge and management gaps among members of the public, people with prediabetes, and healthcare professionals [15]. In China, community‐based work has likewise suggested limited awareness and incomplete intervention among adults with prediabetes [16].

Recent studies have extended this literature but continue to show heterogeneous patterns. A survey of adults in Saudi Arabia identified gaps in prediabetes knowledge and barriers to preventive practice [17], whereas another Saudi study that incorporated objective clinical markers found that knowledge, attitudes, and dietary practices did not necessarily vary in parallel with measured prediabetes risk [18]. A recent United Arab Emirates survey similarly reported differences across knowledge, attitudes, practices, and risk levels [19]. However, evidence remains limited for Chinese adults with the combined cardiometabolic profile of hypertension and abdominal obesity. We therefore assessed prediabetes‐related knowledge, attitudes, and reported practices in this population in Nantong, China. We additionally used structural equation modeling (SEM) to examine whether the observed covariance structure was compatible with a hypothesized KAP model while treating the estimated paths as cross‐sectional statistical associations rather than evidence of temporal ordering or causal mediation.

## 2. Methods

### 2.1. Study Design and Participants

This cross‐sectional study was conducted from April to June 2025 at Nantong Maternity and Child Health Care Hospital. Participants were recruited by convenience sampling. Eligibility required a documented history of hypertension together with abdominal obesity. Hypertension was operationalized as systolic blood pressure ≥ 140 mmHg or diastolic blood pressure ≥ 90 mmHg, or a prior clinical diagnosis [20]. Abdominal obesity was defined as waist circumference ≥ 90 cm in men and ≥ 85 cm in women according to Chinese guidance [21]. Participants with known diabetes or current glucose‐lowering treatment, secondary hypertension, significant hepatic or renal dysfunction, pregnancy, malignancy, or cognitive impairment that prevented questionnaire completion were excluded.

The study did not obtain fasting plasma glucose, oral glucose tolerance testing, or HbA1c specifically for research classification. Consequently, participants were selected on the basis of hypertension, abdominal obesity, and clinical history rather than objectively measured glycemic status. Some participants may therefore have had unrecognized prediabetes, normal glycemia, or undiagnosed diabetes. Throughout the revised manuscript, “elevated‐risk population” refers to the cardiometabolic risk profile created by coexisting hypertension and abdominal obesity and not to confirmed prediabetes.

The study was approved by the Ethics Committee of Nantong Maternal and Child Health Care Hospital (Approval No. Y2024032). All participants provided written informed consent before participation, and the study was conducted in accordance with the Declaration of Helsinki and applicable institutional requirements.

### 2.2. Questionnaire Development and Item Structure

A self‐developed questionnaire was informed by prior diabetes and prediabetes KAP literature [22, 23] and pilot‐tested in 25 participants. The pilot questionnaire had an overall Cronbach′s *α* of 0.878. No test–retest reliability, criterion validity, responsiveness, or independent external validation study was performed; these limitations are considered in the interpretation of the psychometric findings.

The questionnaire contained demographic/clinical questions plus three KAP domains. A separate screening question asked whether the participant had heard of “prediabetes”; this item was not included in the knowledge score or latent model. The scored knowledge domain comprised 12 true/false/not‐sure items (K2–K13), scored 1 for a correct answer and 0 otherwise (range 0–12). The attitude domain comprised nine Likert items (A1–A9; five response categories). The original practice domain comprised six frequency items (P1–P6), scored from 1 to 5. The corrected Appendix S1 provides the exact item mapping.

A revision‐stage data and coding audit identified that P5 (frequency of including whole grains and high‐fiber foods) had been reverse‐scored in the original submission. P5 was corrected so that greater frequency received a higher score, and all analyses involving practice were recomputed. In the corrected full‐item CFA, P4 and P6 had standardized loadings of 0.056 (*p* = 0.378) and −0.068 (*p* = 0.291), respectively. Because these indicators contributed little to the one‐factor practice construct, the primary respecified CFA/SEM used P1, P2, P3, and P5. The primary practice score used for Table 1 and the main correlation analysis was therefore recalculated from these four items (range 4–20). The original six practice items were retained in item‐level descriptive reporting and full‐item sensitivity analyses so that the effect of this data‐driven respecification could be evaluated.

**Table 1:** Demographic characteristics and KAP scores of hypertensive patients with abdominal obesity.

Variables	*N*(%)	Knowledge, mean ± SD	*p*	Attitude, mean ± SD	*p*	Practice, mean ± SD	*p*
*N* = 446		8.9 ± 2.14		37.31 ± 3.68		10.75 ± 2.38	
Gender			0.175		0.596		0.517
Male	222 (49.78)	8.77 ± 2.15		37.25 ± 3.73		10.74 ± 2.43	
Female	224 (50.22)	9.03 ± 2.13		37.38 ± 3.64		10.77 ± 2.34	
Age	65.78 ± 9.39						
BMI			0.627		0.507		0.043
18.5–24.9	167 (37.44)	9.07 ± 2.04		37.01 ± 3.68		11.13 ± 2.4	
25–29.9	226 (50.67)	8.77 ± 2.27		37.5 ± 3.72		10.57 ± 2.30	
≥ 30	53 (11.88)	8.94 ± 1.86		37.43 ± 3.51		10.38 ± 2.67	
Waist circumference	96.52 ± 7.68						
Systolic	142.79 ± 15.55						
Diastolic	84.47 ± 10.24						
Average monthly income			0.002		0.502		0.001
< 2000	55 (12.33)	8.15 ± 2.37		37.2 ± 3.91		9.78 ± 2.65	
2001–5000	248 (55.61)	8.83 ± 2.16		37.15 ± 3.64		10.93 ± 2.31	
> 5000	143 (32.06)	9.31 ± 1.91		37.64 ± 3.68		10.83 ± 2.32	
Education			< 0.001		< 0.001		0.168
Primary school or below	47 (10.54)	7.81 ± 2.34		36 ± 3.36		10.26 ± 2.17	
Junior high school	172 (38.57)	8.7 ± 2.14		36.85 ± 3.67		10.64 ± 2.44	
Senior high school/technical secondary school	158 (35.43)	8.97 ± 2.15		37.43 ± 3.67		10.78 ± 2.29	
Associate/bachelor′s degree or above	69 (15.47)	9.96 ± 1.4		39.07 ± 3.35		11.30 ± 2.53	
Marital status			0.990		0.110		0.021
Unmarried	41 (9.19)	8.88 ± 2.4		38.34 ± 3.62		9.90 ± 2.54	
Married	405 (90.81)	8.9 ± 2.11		37.21 ± 3.68		10.84 ± 2.35	
Hypertension			0.601		0.980		0.563
Yes	385 (86.32)	8.87 ± 2.17		37.29 ± 3.66		10.72 ± 2.36	
Diagnosed with hypertension but not on medication	61 (13.68)	9.08 ± 1.93		37.43 ± 3.83		10.93 ± 2.54	
Family history of diabetes			0.001		0.794		0.008
Yes	119 (26.68)	9.42 ± 1.88		37.27 ± 3.77		11.25 ± 2.2	
No	327 (73.32)	8.71 ± 2.2		37.33 ± 3.66		10.57 ± 2.42	
Smoking			0.492		0.959		0.012
Yes	80 (17.94)	8.79 ± 1.97		37.2 ± 3.72		10.25 ± 2.24	
No	366 (82.06)	8.92 ± 2.18		37.34 ± 3.68		10.86 ± 2.40	
Alcohol			0.080		0.569		0.152
Yes	108 (24.22)	8.69 ± 1.95		37.53 ± 3.88		10.54 ± 2.61	
No	338 (75.78)	8.97 ± 2.19		37.24 ± 3.62		10.82 ± 2.3	

The 80% “positive KAP” cutoff used in the original submission was removed from the revised analysis. This instrument has no validated clinical threshold that establishes 80% of the maximum score as a meaningful boundary, and the revised manuscript therefore treats KAP scores as continuous descriptive measures rather than clinically validated categories.

### 2.3. Data Collection and Quality Control

Paper questionnaires were administered by trained researchers. Completed forms were checked for completeness and internal plausibility before data entry. Questionnaires with implausible demographic/clinical values or uniform responses across an entire KAP section were excluded. Of 497 collected questionnaires, 51 were excluded according to the documented screening process, leaving 446 participants for analysis.

### 2.4. Statistical Analysis

Analyses were performed with SPSS Version 27.0 and Amos Version 26.0 (IBM, Armonk, New York, United States). Continuous variables are summarized as mean ± standard deviation and categorical variables as *n* (%). Because KAP score distributions were not treated as normally distributed, Mann–Whitney *U* or Kruskal–Wallis tests were used for demographic group comparisons. Spearman rank correlations were used to describe pairwise associations among KAP scores.

CFA was used to examine the measurement model. We report standardized factor loadings, average variance extracted (AVE), and composite reliability (CR). For discriminant separation, the square root of each construct′s AVE was compared with the absolute interconstruct correlations. Because AVE and CR are descriptive psychometric diagnostics rather than binary proof of validity, values below commonly used benchmarks were retained and explicitly interpreted as evidence of measurement weakness. Model fit was summarized using CMIN/DF, RMSEA, IFI, TLI, and CFI; the revised analysis used conventional targets of RMSEA < 0.08 and IFI/TLI/CFI > 0.90 rather than the more permissive > 0.80 criterion used in the original manuscript [24, 25].

The structural model evaluated the hypothesized paths knowledge→attitude, attitude→practice, and knowledge→practice. Bootstrap 95% confidence intervals were reported for total, direct, and model‐based indirect effects. Because KAP domains were measured concurrently, the indirect effect is described as an indirect statistical association compatible with the hypothesized model and not as evidence of causal mediation. Sensitivity analyses included (i) a full six‐item practice latent‐variable SEM and (ii) a path model based on full‐item manifest scores. Multicollinearity diagnostics and modification indices were also examined.

No formal a priori power calculation was performed when the study was designed; planning relied on a conventional cases‐per‐indicator rule for SEM. In response to peer review, an RMSEA‐based post hoc sensitivity analysis following MacCallum et al. [26] was added. With *N* = 446, *d* *f* = 321, and *α* = 0.05, the calculated power was approximately 1.00 for distinguishing close fit (RMSEA = 0.05) from poor fit (RMSEA = 0.08), and the corresponding sample size for 80% power was approximately 64. This retrospective calculation describes sensitivity of the global fit test and does not replace an a priori sample‐size justification. Two‐sided *p* < 0.05 was considered statistically significant.

## 3. Results

### 3.1. Participants and KAP scores

Of 497 collected questionnaires, five were excluded for implausible or internally inconsistent values, eight because the participant did not meet the hypertension criterion, and 38 because abdominal obesity was absent. The final sample therefore comprised 446 participants. Mean age was 65.78 ± 9.39 years, and 222 (49.78%) were men. After correction of P5 and respecification of the primary practice score, mean scores were 8.90 ± 2.14 for knowledge, 37.31 ± 3.68 for attitude, and 10.75 ± 2.38 for the four‐item practice score. Demographic comparisons are shown in Table 1. Practice scores differed by BMI (*p* = 0.043), monthly income (*p* = 0.001), marital status (*p* = 0.021), family history of diabetes (*p* = 0.008), and smoking (*p* = 0.012), whereas several differences reported in the original submission were no longer present after the corrected scoring.

### 3.2. Item Distributions and Ceiling Effects

Knowledge was uneven across items. Correct‐response rates were 37.67% for the fasting‐glucose–range item and 29.60% for the item asking whether glucose‐lowering medication should be started immediately, whereas several lifestyle and risk items exceeded 80% correct (Table S1). Attitude responses showed pronounced ceiling effects: 99.77% agreed or strongly agreed that understanding prediabetes is important for diabetes prevention, 96.86% were willing to modify lifestyle, and 99.55% considered regular health checks important (Table S2). These highly concentrated responses limit discrimination among participants and may attenuate associations with other constructs.

For practice, 50.67% reported rarely or never monitoring blood glucose weekly and 76.01% rarely or never read diabetes educational materials weekly (Table S3). These frequencies are descriptive. Because routine weekly glucose monitoring and weekly reading of educational materials are not validated behavioral targets for every person without diagnosed prediabetes, the revised manuscript does not label these responses as guideline‐defined deficiencies.

### 3.3. Measurement Model and Psychometric Findings

The primary respecified CFA included K2–K13, A1–A9, and P1/P2/P3/P5 (Figure 1; Table 2). Standardized loadings for the four practice indicators ranged from 0.236 to 0.648. Attitude indicators were generally strong except for the inversely worded A2 (standardized loading −0.165), whereas several knowledge items had modest loadings. AVE/CR were 0.186/0.699 for knowledge, 0.636/0.936 for attitude, and 0.199/0.464 for practice (Table 3). Thus, the attitude construct showed acceptable convergent reliability, whereas knowledge and especially practice showed weak convergent measurement properties.

**Figure 1:**
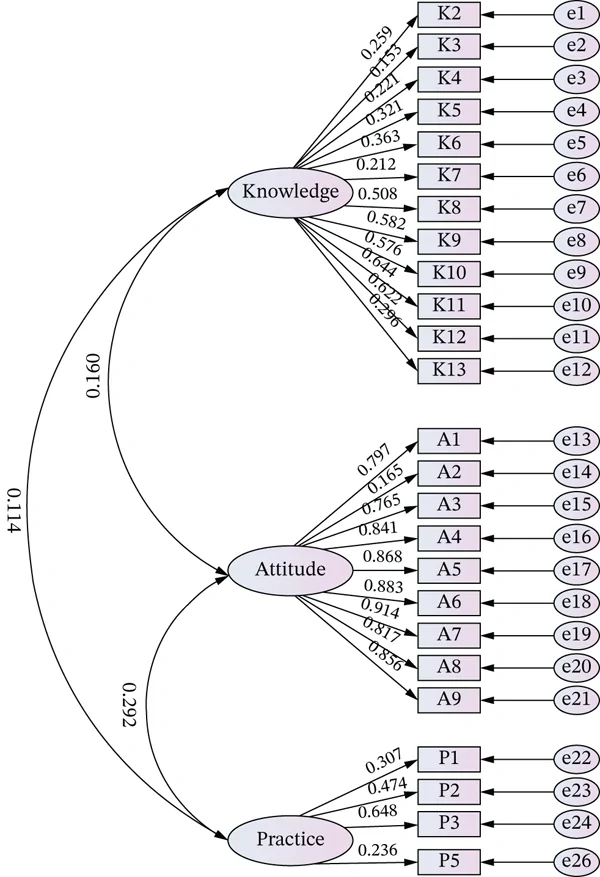
Primary respecified confirmatory factor analysis model after removal of P4 and P6 from the practice construct.

**Table 2:** Primary respecified CFA measurement model.

			Estimate	Standardized estimate	S.E.	C.R.	*p*
K2	←	Knowledge	1.000	0.259			
K3	←	Knowledge	0.587	0.153	0.237	2.474	0.013
K4	←	Knowledge	0.788	0.221	0.248	3.183	0.001
K5	←	Knowledge	0.656	0.321	0.170	3.863	< 0.001
K6	←	Knowledge	1.050	0.363	0.259	4.053	< 0.001
K7	←	Knowledge	0.766	0.212	0.247	3.104	0.002
K8	←	Knowledge	1.360	0.508	0.304	4.468	< 0.001
K9	←	Knowledge	1.833	0.582	0.399	4.593	< 0.001
K10	←	Knowledge	1.272	0.576	0.278	4.584	< 0.001
K11	←	Knowledge	1.439	0.644	0.308	4.666	< 0.001
K12	←	Knowledge	1.232	0.622	0.265	4.642	< 0.001
K13	←	Knowledge	1.069	0.296	0.287	3.722	< 0.001
A9	←	Attitude	1.000	0.856			
A8	←	Attitude	0.983	0.817	0.045	22.068	< 0.001
A7	←	Attitude	1.035	0.914	0.038	27.215	< 0.001
A6	←	Attitude	1.049	0.883	0.041	25.429	< 0.001
A5	←	Attitude	0.991	0.868	0.040	24.613	< 0.001
A4	←	Attitude	0.973	0.841	0.042	23.243	< 0.001
A3	←	Attitude	0.987	0.765	0.050	19.832	< 0.001
A2	←	Attitude	−0.277	−0.165	0.081	−3.439	< 0.001
A1	←	Attitude	0.872	0.797	0.041	21.187	< 0.001
P1	←	Practice	1.000	0.307			
P2	←	Practice	1.166	0.474	0.288	4.046	< 0.001
P3	←	Practice	1.569	0.648	0.408	3.845	< 0.001
P5	←	Practice	0.585	0.236	0.197	2.972	0.003

**Table 3:** Spearman correlations, average variance extracted (AVE), and composite reliability (CR).

Dimensions	Knowledge	Attitude	Practice	AVE	CR
Knowledge	1			0.186	0.699
Attitude	0.160 (*p* < 0.001)	1		0.636	0.936
Practice	0.173 (*p* < 0.001)	0.146 (*p* = 0.002)	1	0.199	0.464

The square roots of AVE were approximately 0.431 for knowledge, 0.797 for attitude, and 0.446 for practice, each exceeding the corresponding absolute interconstruct correlations (0.146–0.173). This pattern supports empirical separation of the three constructs, but it does not offset the low AVE and CR of the knowledge/practice domains. The full six‐item practice CFA confirmed the reviewer′s concern: P4 and P6 had near‐zero nonsignificant loadings, and the full practice construct had AVE = 0.135 and CR = 0.380 (Table S4 and Figure S1).

### 3.4. Correlations Among KAP Scores

After correction of P5 and use of the respecified four‐item practice score, Spearman correlations were significant but small: knowledge–attitude *r* = 0.160 (*p* < 0.001), knowledge–practice *r* = 0.173 (*p* < 0.001), and attitude–practice *r* = 0.146 (*p* = 0.002) (Table 3). The previously reported nonsignificant attitude‐practice correlation (*r* = 0.051) was therefore not reproduced after the scoring correction and model respecification.

### 3.5. Hypothesized Structural Model

The primary SEM showed acceptable global fit (CMIN/DF = 2.127, RMSEA = 0.050, IFI = 0.926, TLI = 0.918, CFI = 0.925; Tables 4 and 5 and Figure 2). Knowledge was associated with attitude (*β* = 0.160, 95% CI 0.049–0.262; *p* = 0.012), and attitude was associated with practice (*β* = 0.281, 95% CI 0.181–0.436; *p* = 0.004). The direct knowledge–practice path was not significant (*β* = 0.069, 95% CI −0.094 to 0.246; *p* = 0.402). The model‐based indirect association from knowledge to practice through attitude was 0.045 (95% CI 0.016–0.091; *p* = 0.004). Because all variables were measured at the same time, this indirect association does not establish temporal mediation.

**Table 4:** Fit indices and structural paths for the primary respecified SEM.

Indicators	Reference	Actual
CMIN/DF	1–3: Excellent, 3–5: good	2.127
RMSEA	< 0.08: Good	0.050
IFI	> 0.9: Good	0.926
TLI	> 0.9: Good	0.918
CFI	> 0.9: Good	0.925

**Table 5:** Standardized total, direct, and indirect effects.

Model paths	Standardized total effects		Standardized direct effects		Standardized indirect effects	
	*β*(95% CI)	*p*	*β*(95% CI)	*p*	*β*(95% CI)	*p*
Knowledge→attitude	0.160 (0.049, 0.262)	0.012	0.160 (0.049, 0.262)	0.012		
Knowledge→practice	0.114 (−0.060, 0.288)	0.180	0.069 (−0.094, 0.246)	0.402		
Attitude→practice	0.281 (0.181, 0.436)	0.004	0.281 (0.181, 0.436)	0.004		
Knowledge→practice					0.045 (0.016, 0.091)	0.004

**Figure 2:**
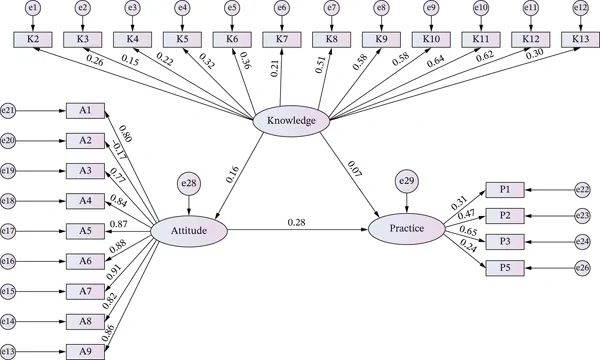
Primary latent‐variable structural equation model. Coefficients shown are standardized estimates.

### 3.6. Sensitivity Analyses and Model Diagnostics

In the full‐item latent SEM, which retained P4 and P6 after correction of P5, fit remained similar (CMIN/DF = 2.050, RMSEA = 0.049, IFI = 0.919, TLI = 0.911, CFI = 0.919). The knowledge–attitude and attitude–practice paths remained statistically significant, whereas the direct knowledge–practice path remained nonsignificant (Table S5 and Figure S2). In the manifest full‐item path analysis, knowledge–attitude *β* = 0.152 (*p* = 0.018), knowledge–practice direct *β* = 0.120 (*p* = 0.006), and attitude–practice *β* = 0.099 (*p* = 0.050); the indirect association was 0.015 (*p* = 0.032) (Table 6 and Figure 3). The attenuation of some coefficients across specifications reinforces that the estimated relationships are sensitive to measurement choices. VIFs were close to 1, providing no evidence of problematic multicollinearity (Table S6). Modification indices are reported in Table S7.

**Table 6:** Sensitivity analysis using full‐item manifest KAP scores.

Model paths	Standardized total effects		Standardized direct effects		Standardized indirect effects	
	*β*(95% CI)	*p*	*β*(95% CI)	*p*	*β*(95% CI)	*p*
Knowledge→attitude	0.152 (0.044, 0.225)	0.018	0.152 (0.044, 0.225)	0.018		
Knowledge→practice	0.136 (0.049, 0.225)	0.007	0.120 (0.041, 0.209)	0.006		
Attitude→practice	0.099 (0.000, 0.190)	0.050	0.099 (0.000, 0.190)	0.050		
Knowledge→practice					0.015 (0.002, 0.032)	0.032

**Figure 3:**
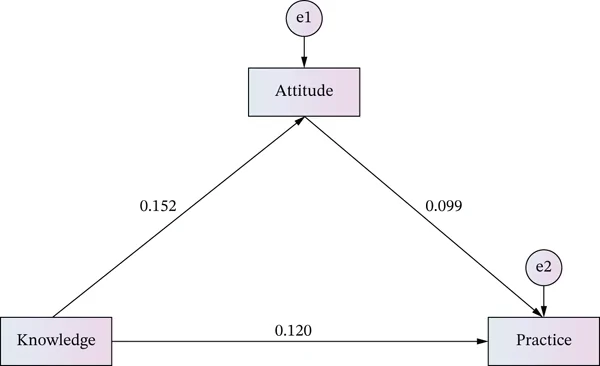
Sensitivity path analysis based on full‐item manifest KAP scores. Coefficients shown are standardized estimates.

## 4. Discussion

In this cross‐sectional sample of adults with hypertension and abdominal obesity, prediabetes‐related knowledge, attitudes, and reported practices were modestly associated. After correcting P5 scoring and removing P4/P6 from the primary practice measurement model, the attitude‐practice correlation was small but statistically significant, and the latent SEM showed associations from knowledge to attitude and from attitude to practice. A statistically significant indirect knowledge–practice association was also observed. These findings are compatible with the hypothesized KAP framework, but the concurrent measurement of all domains means that neither temporal ordering nor causal mediation can be inferred. Equally important, the revised psychometric analysis identified substantial measurement limitations, particularly for the practice construct.

The item‐level findings are broadly consistent with the heterogeneous picture reported in prediabetes KAP research. A systematic review found recurrent deficits in prediabetes knowledge and management across the public, people with prediabetes, and healthcare professionals [15]. Earlier community‐based work in China also suggested that awareness and intervention among adults with prediabetes were incomplete [16]. In our sample, knowledge was relatively strong for several general lifestyle and risk statements but substantially weaker for glucose thresholds and treatment‐related items. A recent Saudi survey similarly identified knowledge gaps and barriers despite generally favorable views toward prevention [17]. Another Saudi study using objective clinical markers showed that knowledge, attitudes, dietary practices, and measured risk did not necessarily move together [18], whereas a United Arab Emirates survey also demonstrated variation across these domains [19]. Taken together, these comparisons support interpreting KAP as a set of related but nonequivalent constructs rather than as a single continuum of preventive readiness.

The attitude results deserve separate caution because several items showed very little variation. Nearly all participants endorsed the importance of understanding prediabetes, lifestyle modification, and regular health checks, producing a pronounced ceiling effect. Similar KAP studies in other diabetes‐related populations have shown that favorable attitudes can coexist with incomplete knowledge or inconsistent self‐reported behavior. For example, a population‐based study in rural Bangladesh reported substantial gaps across diabetes knowledge and practice despite areas of positive attitude [27], and work in patients with Type 2 diabetes in Benin also found uneven KAP profiles [28]. A national/resident survey in Qatar likewise demonstrated that the three domains were not uniformly aligned [29]. Chinese studies also illustrate heterogeneity within related high‐risk groups: Negative emotions have been documented among community‐dwelling adults with prediabetes and hypertension [30], whereas abdominal obesity is common among middle‐aged and older adults with hypertension [31]. In our data, restricted variation in attitude items probably reduced discrimination among participants and may have attenuated observed correlations. It may also reflect social‐desirability effects, so the high proportion of favorable responses should not be interpreted as evidence that attitudes were optimally measured or that favorable attitudes necessarily translate into behavior.

The practice domain requires even more cautious interpretation. Its original six items cover heterogeneous behaviors, exercise, glucose monitoring, reading educational materials, alcohol consumption, whole‐grain/high‐fiber intake, and distracted eating, so a single latent factor is a simplifying assumption rather than a demonstrated unidimensional behavioral construct. The reviewer correctly identified extremely weak loadings for P4 and P6. Respecifying the primary model without these items improved focus, but the four‐item practice construct still had AVE = 0.199 and CR = 0.464, indicating weak convergent measurement properties. Behavioral determinants in prediabetes are likely to be multidimensional; a theory of planned behavior study, for example, identified distinct psychosocial determinants of lifestyle behavior rather than a single undifferentiated practice tendency [32]. The present sensitivity analyses showed similar direction for some structural paths but smaller or specification‐dependent coefficients when all practice items were retained. The structural findings should therefore be regarded as exploratory and contingent on measurement choices rather than as validation of a stable behavioral pathway.

The revised descriptive interpretation also distinguishes low‐frequency behaviors from clinically inappropriate behaviors. Routine weekly self‐monitoring of blood glucose is not necessarily indicated for every person without diagnosed dysglycemia, and weekly reading of diabetes educational materials is not an established universal target. We therefore describe these response frequencies without labeling them as “deficiencies.” Their value is primarily descriptive and hypothesis‐generating. Previous prediabetes education work has shown that knowledge and reported practices can change after a structured program [22], but such evidence does not establish that the specific weekly behaviors used in our questionnaire are validated targets. Future instruments should therefore define practice items against population‐appropriate recommendations and, where possible, separate dietary, physical activity, monitoring, and healthcare‐seeking behaviors.

The apparent discrepancy between the original simple correlation and SEM path was also resolved empirically during revision. The original manuscript reported an attitude‐practice correlation of *r* = 0.051 (*p* = 0.286) but a significant latent path. After correction of P5 scoring and respecification of the practice construct, the corresponding Spearman correlation was *r* = 0.146 (*p* = 0.002), whereas the latent path was *β* = 0.281 (*p* = 0.004). These estimates are not expected to be numerically identical because SEM estimates associations among latent variables under a specified measurement model, whereas Spearman correlation describes an unadjusted association between observed scores. The coding and model audit therefore reduced the apparent contradiction, but the modest effect sizes and sensitivity to model specification remain important. The indirect coefficient should likewise be interpreted only as a model‐based cross‐sectional association compatible with the hypothesized KAP structure, not as formal mediation.

The findings may nevertheless help define questions for prospective research. Large prevention trials have established that lifestyle modification can reduce progression to T2DM in people with impaired glucose regulation [5, 6], and contemporary clinical reviews emphasize structured weight, diet, and physical activity management in prediabetes [11]. Our results do not demonstrate that education will improve behavior or reduce diabetes incidence, but they suggest specific areas—such as understanding glycemic thresholds and treatment expectations—that could be tested as intervention targets. Studies of prediabetes education [22] and behavioral determinants [32] provide useful precedents for developing more focused interventions, whereas research among general practitioners also underscores that prediabetes knowledge and management gaps can occur on the healthcare‐provider side as well as among patients [33]. Future studies should predefine behavioral outcomes, use externally validated or newly validated multidimensional instruments, and include objective HbA1c, fasting glucose, or oral glucose tolerance testing so that participants with confirmed prediabetes can be distinguished from those who are simply at elevated cardiometabolic risk.

Several limitations are important. First, the cross‐sectional design prevents temporal or causal inference and does not support formal mediation claims. Second, convenience sampling from a single center can produce selection bias; people attending clinical services may have greater health awareness than the broader hypertensive population, limiting external validity. Third, the questionnaire was self‐developed. Although pilot internal consistency and CFA were assessed, test–retest reliability, criterion validity, responsiveness, and independent external validation were not evaluated, and convergent reliability was weak for knowledge and practice. Fourth, all principal outcomes were self‐reported, creating potential recall and social‐desirability bias; the strong attitude ceiling effect is consistent with this concern. Fifth, no study‐specific glucose or HbA1c testing was performed, so actual prediabetes prevalence was unknown. Sixth, potentially important confounders, including duration of hypertension, specific antihypertensive therapies, previous nutrition counseling, healthcare utilization, urban/rural residence, prior diabetes screening, and more detailed cognitive assessment, were not measured comprehensively. Seventh, removal of P4/P6 was data‐driven and should be externally replicated. Finally, no a priori SEM power calculation was performed; the added RMSEA analysis is retrospective and should not be interpreted as replacing prospective sample‐size planning.

## 5. Conclusion

This cross‐sectional study suggests small associations among prediabetes‐related knowledge, attitudes, and reported practices in adults with hypertension and abdominal obesity. A respecified SEM was compatible with a hypothesized KAP structure, including a model‐based indirect association, but the data do not establish directionality or mediation. Measurement limitations, especially weak convergent reliability of the practice construct, a strong attitude ceiling effect, self‐report, and the absence of objective glycemic classification, substantially constrain interpretation. Longitudinal and externally validated studies are required before behavioral pathways or intervention strategies can be inferred.

## Author Contributions

**Jian Chen:** conceptualization, data curation, validation, writing – original draft, writing – review and editing. **Xia Chao:** conceptualization, investigation, writing – original draft. **Jinyun Zhang:** methodology, formal analysis, software, writing – original draft, writing – review and editing. **Liting Zhou:** methodology, formal analysis, resources, writing – original draft. **Bofan Huang:** funding acquisition, project administration, supervision, writing – review and editing.

## Funding

This study was funded by the Major Collaborative Innovation Project of Medical and Health Science and Technology Innovation (2021‐I2M‐1‐009).

## Disclosure

All authors have read and approved the final version of the manuscript. Jian Chen, the corresponding author and manuscript guarantor, had full access to all of the data in this study and takes complete responsibility for the integrity of the data and the accuracy of the data analysis.

## Ethics Statement

The study was approved by the Ethics Committee of Nantong Maternal and Child Health Care Hospital (Approval No. Y2024032) and was conducted in accordance with the Declaration of Helsinki and applicable institutional requirements.

## Consent

All participants provided written informed consent before enrollment. Consent for publication was not acquired as the manuscript does not contain identifiable individual‐level information.

## Conflicts of Interest

The authors declare no conflicts of interest.

## Supporting information


**Supporting Information** Additional supporting information can be found online in the Supporting Information section. Appendix S1: Questionnaire. Table S1: Distribution of knowledge responses. Table S2: Distribution of attitude responses. Table S3: Distribution of practice responses (six original practice items). Table S4: Corrected full‐item CFA model, including all original practice indicators. Table S5: Full‐item latent‐variable SEM sensitivity analysis. Table S6: Multicollinearity diagnostics and RMSEA‐based post hoc sensitivity analysis. Table S7: Modification indices for the primary SEM model. Figure S1: Sensitivity analysis of the full‐item confirmatory factor analysis (CFA) measurement model. Figure S2: Sensitivity analysis of the full‐item latent‐variable structural equation model (SEM).

## Data Availability

All data generated or analyzed during this study are included in this published article.
