# Supplementary material for: Knowledge, Attitudes, and Reported Practices Regarding Prediabetes Among Hypertensive Patients With Abdominal Obesity: A Cross‐Sectional Structural Equation Modeling Analysis
Source: J Diabetes Res. 2026 Oct 10;2026:3315859. doi: 10.1155/jdr/3315859 (PMC13653257; doi:10.1155/jdr/3315859)
Supplement: Supplementary file 1 [file JDR-2026-3315859-s001.zip › JDR_3315859_Supplementary_Material.docx]

**Supplementary Tables**

Supplementary Tables S1–S3 present item-level response distributions, whereas Supplementary Tables S4–S7 present the corrected psychometric, sensitivity, and model-diagnostic analyses. P5 scoring was corrected during revision. P4 and P6 are retained in the full-item sensitivity analyses but excluded from the primary re-specified practice measurement model because their standardized loadings were near zero and nonsignificant.

**Supplementary Table S1. Distribution of knowledge responses.**

| **Knowledge dimension items** | **Yes/True, n (%)** | **No/False, n (%)** | **Not sure, n (%)** | **Correct response, n (%)** |
| --- | --- | --- | --- | --- |
| Are you aware of the concept of "prediabetes"? | 315 (70.63) | 131 (29.37) |  |  |
| Prediabetes refers to blood sugar levels higher than normal but below the diagnostic threshold for diabetes. | 272 (60.99) | 14 (3.14) | 160 (35.87) | 272 (60.99) |
| Fasting blood glucose levels between 5.6-6.9 mmol/L fall within the normal range. | 191 (42.83) | 168 (37.67) | 87 (19.51) | 168 (37.67) |
| Prediabetes requires no intervention. | 67 (15.02) | 319 (71.52) | 60 (13.45) | 319 (71.52) |
| Individuals with prediabetes need regular blood glucose monitoring. | 414 (92.83) | 11 (2.47) | 21 (4.71) | 414 (92.83) |
| Prediabetes increases the risk of cardiovascular disease. | 375 (84.08) | 6 (1.35) | 65 (14.57) | 375 (84.08) |
| Hypertensive patients with abdominal obesity diagnosed with prediabetes should immediately start glucose-lowering medication. | 124 (27.8) | 132 (29.6) | 190 (42.6) | 132 (29.6) |
| Blood tests are typically recommended for detecting prediabetes. | 387 (86.77) | 7 (1.57) | 52 (11.66) | 387 (86.77) |
| Family history of diabetes, abdominal obesity, advanced age, and long-term high-sugar diets are associated with increased risk of prediabetes. | 358 (80.27) | 8 (1.79) | 80 (17.94) | 358 (80.27) |
| Regular physical activity, healthy diet, reduced salt intake, and periodic health check-ups can help hypertensive patients with abdominal obesity lower their risk of prediabetes. | 408 (91.48) | 7 (1.57) | 31 (6.95) | 408 (91.48) |
| Prediabetes increases the risk of cerebrovascular diseases and diabetes. | 407 (91.26) | 3 (0.67) | 36 (8.07) | 407 (91.26) |
| Adequate intake of high-fiber foods and fresh fruits/vegetables helps control blood sugar and weight, thereby aiding prediabetes management. | 416 (93.27) | 2 (0.45) | 28 (6.28) | 416 (93.27) |
| Prediabetes may present with non-specific symptoms like dry mouth, blurred vision, weight loss, or no symptoms at all. | 313 (70.18) | 22 (4.93) | 111 (24.89) | 313 (70.18) |

Note: The awareness item is a screening question and was not included in the scored knowledge domain; therefore, no correctness rate is reported for that item.

**Supplementary Table S2. Distribution of attitude responses.**

| **Attitude dimension items** | **Strongly agree, n (%)** | **Agree, n (%)** | **Neutral, n (%)** | **Disagree, n (%)** | **Strongly disagree, n (%)** |
| --- | --- | --- | --- | --- | --- |
| I believe understanding prediabetes is crucial for preventing diabetes. | 236 (52.91) | 209 (46.86) | 1 (0.22) |  |  |
| I think prediabetes does not require special attention. | 10 (2.24) | 20 (4.48) | 18 (4.04) | 320 (71.75) | 78 (17.49) |
| I am willing to modify my lifestyle to prevent prediabetes. | 171 (38.34) | 261 (58.52) | 8 (1.79) | 5 (1.12) | 1 (0.22) |
| I consider regular health check-ups important for early detection of prediabetes. | 193 (43.27) | 251 (56.28) |  | 1 (0.22) | 1 (0.22) |
| I believe individuals with prediabetes should seek guidance from healthcare professionals. | 188 (42.15) | 250 (56.05) | 8 (1.79) |  |  |
| If diagnosed with prediabetes, I would actively take measures to control my blood sugar. | 192 (43.05) | 245 (54.93) | 7 (1.57) | 2 (0.45) |  |
| I think dietary modifications are essential for managing prediabetes. | 198 (44.39) | 244 (54.71) | 3 (0.67) | 1 (0.22) |  |
| I believe increased physical activity can effectively prevent prediabetes. | 184 (41.26) | 250 (56.05) | 10 (2.24) | 2 (0.45) |  |
| I would undergo regular prediabetes screening if recommended by a doctor. | 180 (40.36) | 258 (57.85) | 6 (1.35) | 2 (0.45) |  |

**Supplementary Table S3. Distribution of practice responses (six original practice items).**

| **Practice dimension items** | **Never, n (%)** | **Rarely, n (%)** | **Occasionally, n (%)** | **Frequently, n (%)** | **Always, n (%)** |
| --- | --- | --- | --- | --- | --- |
| How often do you engage in moderate-intensity exercise weekly? (e.g., brisk walking, swimming) | 53 (11.88) | 123 (27.58) | 118 (26.46) | 108 (24.22) | 44 (9.87) |
| How often do you monitor your blood glucose weekly? | 45 (10.09) | 181 (40.58) | 170 (38.12) | 37 (8.3) | 13 (2.91) |
| How often do you read educational materials about diabetes weekly? | 142 (31.84) | 197 (44.17) | 83 (18.61) | 20 (4.48) | 4 (0.9) |
| How often do you consume alcoholic beverages weekly? | 260 (58.3) | 91 (20.4) | 34 (7.62) | 32 (7.17) | 29 (6.5) |
| How often do you include whole grains and high-fiber foods in your weekly diet? | 5 (1.12) | 77 (17.26) | 179 (40.13) | 145 (32.51) | 40 (8.97) |
| How often do you eat distractedly while watching TV/using your phone weekly? | 126 (28.25) | 169 (37.89) | 117 (26.23) | 31 (6.95) | 3 (0.67) |

**Supplementary Table S4. Corrected full-item CFA model, including all original practice indicators.**

| **Indicator** | **Latent construct** | **Estimate** | **Standardized estimate** | **S.E.** | **C.R.** | **P** | **AVE** | **CR** |
| --- | --- | --- | --- | --- | --- | --- | --- | --- |
| K2 | Knowledge | 1.000 | 0.260 |  |  |  | 0.187 | 0.699 |
| K3 | Knowledge | 0.587 | 0.153 | 0.237 | 2.476 | 0.013 | 0.187 | 0.699 |
| K4 | Knowledge | 0.787 | 0.221 | 0.247 | 3.184 | 0.001 | 0.187 | 0.699 |
| K5 | Knowledge | 0.655 | 0.321 | 0.169 | 3.865 | <0.001 | 0.187 | 0.699 |
| K6 | Knowledge | 1.050 | 0.363 | 0.259 | 4.057 | <0.001 | 0.187 | 0.699 |
| K7 | Knowledge | 0.766 | 0.212 | 0.246 | 3.108 | 0.002 | 0.187 | 0.699 |
| K8 | Knowledge | 1.358 | 0.508 | 0.304 | 4.472 | <0.001 | 0.187 | 0.699 |
| K9 | Knowledge | 1.832 | 0.583 | 0.399 | 4.597 | <0.001 | 0.187 | 0.699 |
| K10 | Knowledge | 1.270 | 0.576 | 0.277 | 4.587 | <0.001 | 0.187 | 0.699 |
| K11 | Knowledge | 1.437 | 0.644 | 0.308 | 4.670 | <0.001 | 0.187 | 0.699 |
| K12 | Knowledge | 1.230 | 0.622 | 0.265 | 4.646 | <0.001 | 0.187 | 0.699 |
| K13 | Knowledge | 1.069 | 0.296 | 0.287 | 3.726 | <0.001 | 0.187 | 0.699 |
| A1 | Attitude | 1.000 | 0.797 |  |  |  | 0.636 | 0.936 |
| A2 | Attitude | -0.318 | -0.165 | 0.093 | -3.429 | <0.001 | 0.636 | 0.936 |
| A3 | Attitude | 1.133 | 0.765 | 0.062 | 18.159 | <0.001 | 0.636 | 0.936 |
| A4 | Attitude | 1.117 | 0.841 | 0.054 | 20.669 | <0.001 | 0.636 | 0.936 |
| A5 | Attitude | 1.137 | 0.868 | 0.053 | 21.614 | <0.001 | 0.636 | 0.936 |
| A6 | Attitude | 1.203 | 0.883 | 0.054 | 22.160 | <0.001 | 0.636 | 0.936 |
| A7 | Attitude | 1.187 | 0.914 | 0.051 | 23.313 | <0.001 | 0.636 | 0.936 |
| A8 | Attitude | 1.128 | 0.817 | 0.057 | 19.829 | <0.001 | 0.636 | 0.936 |
| A9 | Attitude | 1.147 | 0.856 | 0.054 | 21.189 | <0.001 | 0.636 | 0.936 |
| P1 | Practice | 1.000 | 0.298 |  |  |  | 0.135 | 0.380 |
| P2 | Practice | 1.176 | 0.464 | 0.294 | 4.005 | <0.001 | 0.135 | 0.380 |
| P3 | Practice | 1.663 | 0.667 | 0.439 | 3.785 | <0.001 | 0.135 | 0.380 |
| P4 | Practice | 0.195 | 0.056 | 0.223 | 0.875 | 0.378 | 0.135 | 0.380 |
| P5 | Practice | 0.589 | 0.231 | 0.201 | 2.929 | 0.003 | 0.135 | 0.380 |
| P6 | Practice | -0.181 | -0.068 | 0.171 | -1.064 | 0.291 | 0.135 | 0.380 |

**Supplementary Table S5. Full-item latent-variable SEM sensitivity analysis.**

**Panel A. Model fit indices.**

| **Indicators** | **Reference** | **Actual** |
| --- | --- | --- |
| CMIN/DF | 1-3 excellent，3-5 good | 2.050 |
| RMSEA | <0.08 good | 0.049 |
| IFI | >0.90: Good | 0.919 |
| TLI | >0.90: Good | 0.911 |
| CFI | >0.90: Good | 0.919 |

Panel B. Standardized total, direct, and indirect effects.

| **Model paths** | **Standardized total effects** | | **Standardized direct effects** | | **Standardized indirect effects** | |
| --- | --- | --- | --- | --- | --- | --- |
|  | **β (95% CI)** | **P** | **β (95% CI)** | **P** | **β (95% CI)** | **P** |
| Knowledge→Attitude | 0.160 (0.049,0.260) | 0.012 | 0.160 (0.049,0.260) | 0.012 |  |  |
| Knowledge→Practice | 0.119 (-0.036,0.292) | 0.122 | 0.074 (-0.071,0.253) | 0.319 |  |  |
| Attitude→Practice | 0.281 (0.174,0.453) | 0.003 | 0.281 (0.174,0.453) | 0.003 |  |  |
| Knowledge→Practice |  |  |  |  | 0.045 (0.016-0.098) | 0.003 |

**Panel C. Regression weights for the full-item latent-variable SEM.**

| **Outcome/indicator** | **Relation** | **Predictor/latent construct** | **Estimate** | **Standardized estimate** | **S.E.** | **C.R.** | **P** |
| --- | --- | --- | --- | --- | --- | --- | --- |
| Attitude | <--- | Knowledge | 0.583 | 0.160 | 0.232 | 2.508 | 0.012 |
| Practice | <--- | Attitude | 0.213 | 0.281 | 0.068 | 3.128 | 0.002 |
| Practice | <--- | Knowledge | 0.205 | 0.074 | 0.209 | 0.980 | 0.327 |
| K2 | <--- | Knowledge | 1.000 | 0.260 |  |  |  |
| K3 | <--- | Knowledge | 0.587 | 0.153 | 0.237 | 2.476 | 0.013 |
| K4 | <--- | Knowledge | 0.787 | 0.221 | 0.247 | 3.184 | 0.001 |
| K5 | <--- | Knowledge | 0.655 | 0.321 | 0.169 | 3.865 | <0.001 |
| K6 | <--- | Knowledge | 1.050 | 0.363 | 0.259 | 4.057 | <0.001 |
| K7 | <--- | Knowledge | 0.766 | 0.212 | 0.246 | 3.108 | 0.002 |
| K8 | <--- | Knowledge | 1.358 | 0.508 | 0.304 | 4.472 | <0.001 |
| K9 | <--- | Knowledge | 1.832 | 0.583 | 0.399 | 4.597 | <0.001 |
| K10 | <--- | Knowledge | 1.270 | 0.576 | 0.277 | 4.587 | <0.001 |
| K11 | <--- | Knowledge | 1.437 | 0.644 | 0.308 | 4.670 | <0.001 |
| K12 | <--- | Knowledge | 1.230 | 0.622 | 0.265 | 4.646 | <0.001 |
| K13 | <--- | Knowledge | 1.069 | 0.296 | 0.287 | 3.726 | <0.001 |
| A9 | <--- | Attitude | 1.000 | 0.856 |  |  |  |
| A8 | <--- | Attitude | 0.983 | 0.817 | 0.045 | 22.068 | <0.001 |
| A7 | <--- | Attitude | 1.035 | 0.914 | 0.038 | 27.215 | <0.001 |
| A6 | <--- | Attitude | 1.049 | 0.883 | 0.041 | 25.430 | <0.001 |
| A5 | <--- | Attitude | 0.991 | 0.868 | 0.040 | 24.614 | <0.001 |
| A4 | <--- | Attitude | 0.973 | 0.841 | 0.042 | 23.245 | <0.001 |
| A3 | <--- | Attitude | 0.987 | 0.765 | 0.050 | 19.833 | <0.001 |
| A2 | <--- | Attitude | -0.277 | -0.165 | 0.081 | -3.439 | <0.001 |
| A1 | <--- | Attitude | 0.872 | 0.797 | 0.041 | 21.189 | <0.001 |
| P1 | <--- | Practice | 1.000 | 0.298 |  |  |  |
| P2 | <--- | Practice | 1.176 | 0.463 | 0.294 | 4.005 | <0.001 |
| P3 | <--- | Practice | 1.663 | 0.667 | 0.439 | 3.785 | <0.001 |
| P4 | <--- | Practice | 0.195 | 0.056 | 0.223 | 0.875 | 0.382 |
| P5 | <--- | Practice | 0.589 | 0.230 | 0.201 | 2.929 | 0.003 |
| P6 | <--- | Practice | -0.181 | -0.068 | 0.171 | -1.064 | 0.287 |

*Note: Panel C reports AMOS regression weights and critical-ratio tests, whereas Panel B reports standardized effects with bootstrap confidence intervals; minor P-value differences therefore reflect different output summaries. With P4 and P6 retained, Knowledge→Attitude and Attitude→Practice remained significant, whereas the direct Knowledge→Practice path remained nonsignificant.*

**Supplementary Table S6. Multicollinearity diagnostics and RMSEA-based post hoc sensitivity analysis.**

**Panel A. Multicollinearity diagnostics.**

| **Predictors** | **VIF** | **Tolerance** | **Latent VIF** |
| --- | --- | --- | --- |
| K-A | 1.024 | 0.977 | 1.026 |
| K-P | 1.024 | 0.977 | 1.014 |
| A-P | 1.019 | 0.982 | 1.093 |

Panel B. RMSEA-based post hoc sensitivity analysis.

| **Scenario** | **H0 RMSEA** | **H1 RMSEA** | **α** | **df** | **N** | **Power** | **N for 80%** |
| --- | --- | --- | --- | --- | --- | --- | --- |
| Exact-fit rejection | 0.000 | 0.049 | 0.050 | 321 | 446 | 1.000 |  |
| Close-vs-poor fit discrimination | 0.050 | 0.080 | 0.050 | 321 | 446 | 1.000 | 64 |

Note: This retrospective analysis quantifies sensitivity of global SEM fit testing and does not replace an a priori power calculation.

**Supplementary Table S7. Modification indices for the primary SEM model.**

*Note: The archived AMOS output contained modification indices, whereas the standardized residual covariance matrix was not retained and could not be recovered retrospectively after verification with the statistician. Supplementary Table S7 therefore reports the available modification indices only.*

| **Parameter 1** | **Relation** | **Parameter 2** | **M.I.** | **Par change** |
| --- | --- | --- | --- | --- |
| e22 | <--> | e26 | 7.123 | 0.128 |
| e20 | <--> | Knowledge | 7.706 | -0.014 |
| e20 | <--> | e21 | 5.054 | 0.026 |
| e19 | <--> | e22 | 4.359 | 0.045 |
| e19 | <--> | e21 | 5.689 | -0.014 |
| e18 | <--> | e24 | 4.451 | 0.025 |
| e18 | <--> | e23 | 6.483 | -0.031 |
| e15 | <--> | e29 | 5.408 | -0.013 |
| e15 | <--> | e24 | 7.656 | -0.025 |
| e15 | <--> | e19 | 6.002 | -0.011 |
| e14 | <--> | e19 | 4.242 | 0.013 |
| e14 | <--> | e15 | 6.390 | 0.010 |
| e13 | <--> | e22 | 13.685 | -0.060 |
| e13 | <--> | e16 | 7.956 | 0.011 |
| e12 | <--> | e28 | 9.609 | 0.030 |
| e12 | <--> | e29 | 5.079 | 0.023 |
| e12 | <--> | e24 | 8.050 | 0.048 |
| e10 | <--> | e28 | 6.219 | -0.013 |
| e10 | <--> | e29 | 8.406 | -0.016 |
| e10 | <--> | e22 | 7.633 | -0.035 |
| e10 | <--> | e20 | 7.392 | -0.023 |
| e10 | <--> | e16 | 8.733 | -0.009 |
| e10 | <--> | e11 | 5.080 | 0.005 |
| e9 | <--> | e11 | 7.427 | 0.007 |
| e8 | <--> | e20 | 4.862 | 0.027 |
| e8 | <--> | e12 | 9.990 | 0.023 |
| e7 | <--> | e21 | 8.820 | -0.014 |
| e6 | <--> | e29 | 6.102 | 0.025 |
| e6 | <--> | e16 | 11.967 | -0.021 |
| e6 | <--> | e11 | 5.125 | -0.010 |
| e5 | <--> | e29 | 4.084 | 0.016 |
| e5 | <--> | e11 | 9.715 | -0.011 |
| e4 | <--> | e18 | 4.162 | -0.007 |
| e4 | <--> | e11 | 4.281 | -0.005 |
| e4 | <--> | e8 | 9.941 | -0.013 |
| e4 | <--> | e7 | 9.524 | 0.011 |
| e3 | <--> | e22 | 4.198 | 0.049 |
| e3 | <--> | e21 | 4.817 | -0.015 |
| e3 | <--> | e20 | 21.609 | -0.074 |
| e3 | <--> | e17 | 4.373 | -0.012 |
| e3 | <--> | e14 | 7.994 | 0.020 |
| e3 | <--> | e8 | 5.130 | -0.016 |
| e3 | <--> | e6 | 4.074 | 0.019 |
| e3 | <--> | e4 | 29.928 | 0.028 |
| e2 | <--> | e17 | 6.334 | -0.016 |
| e2 | <--> | e6 | 18.133 | 0.043 |
| e2 | <--> | e4 | 4.755 | 0.012 |
| e2 | <--> | e3 | 9.393 | 0.031 |
| e1 | <--> | e28 | 6.137 | 0.026 |
| e1 | <--> | e21 | 8.682 | 0.021 |
| e1 | <--> | e15 | 5.021 | 0.012 |
| e1 | <--> | e13 | 7.325 | -0.018 |
| e1 | <--> | e12 | 9.980 | 0.031 |
| e1 | <--> | e10 | 5.261 | -0.012 |
| e1 | <--> | e8 | 4.827 | 0.017 |
| e1 | <--> | e6 | 5.247 | 0.023 |

**Supplementary Figure Legends**

**Figure S1. Sensitivity analysis of the full-item confirmatory factor analysis (CFA) measurement model.** This model incorporates all six original practice items (P1–P6) to evaluate measurement properties prior to removing P4 and P6. Standardized factor loadings and inter-construct correlations are displayed.

**Figure S2. Sensitivity analysis of the full-item latent-variable structural equation model (SEM).** This structural model retains the complete six-item practice construct (including P4 and P6). Standardized path estimates are shown (Knowledge → Attitude β = 0.16, Attitude → Practice β = 0.28, direct Knowledge → Practice β = 0.07).
