# Supplementary material for: Knowledge, Attitudes, and Reported Practices Regarding Prediabetes Among Hypertensive Patients With Abdominal Obesity: A Cross‐Sectional Structural Equation Modeling Analysis
Source: J Diabetes Res. 2026 Oct 10;2026:3315859. doi: 10.1155/jdr/3315859 (PMC13653257; doi:10.1155/jdr/3315859)
Supplement: Supplementary file 1 [file JDR-2026-3315859-s001.zip › Figure S2.pdf]

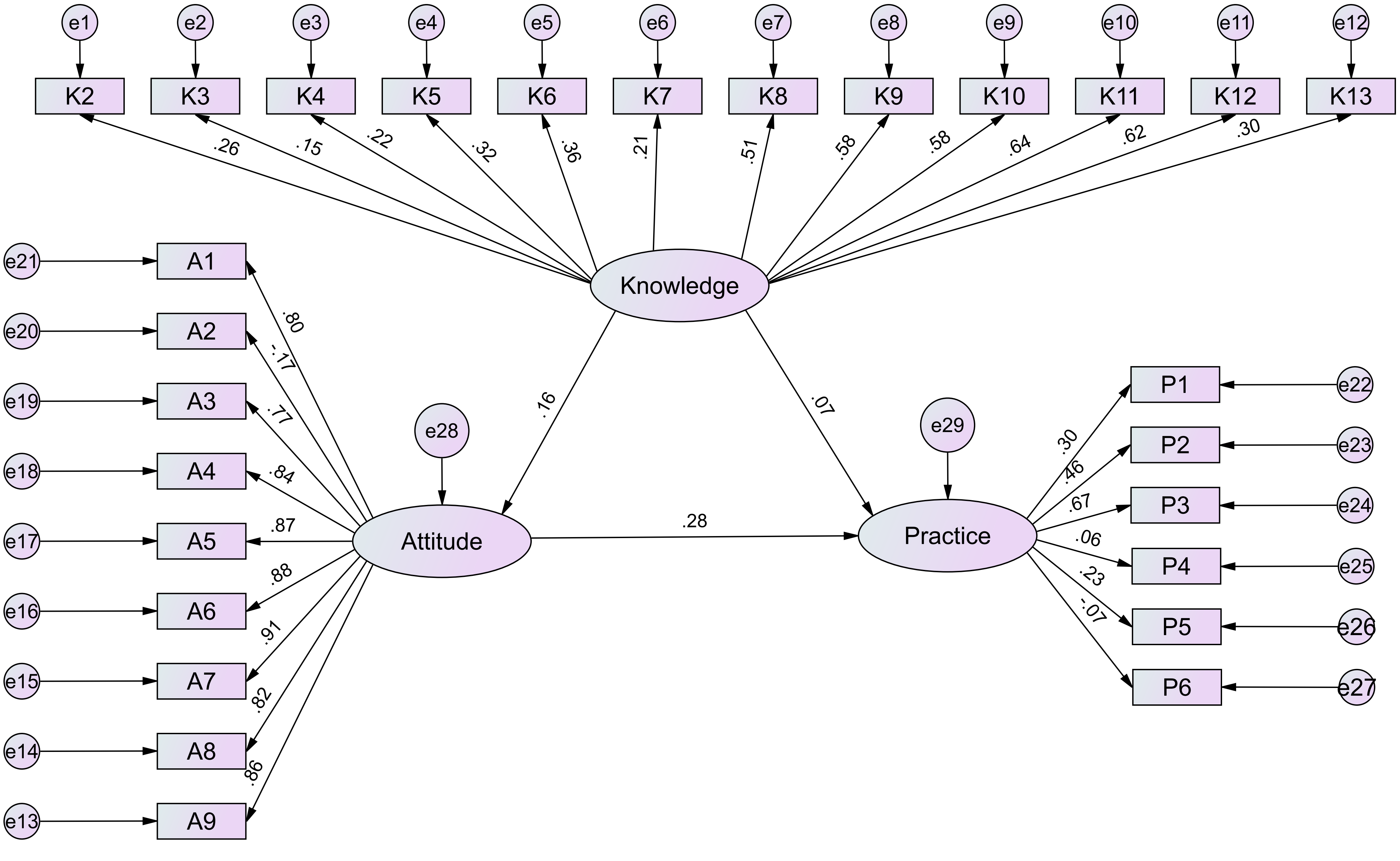

e1
e2
e3
e4
e5
e6
e7
e8
e9
e10
e11
e12
K2
K3
K4
K5
K6
K7
K8
K9
K10
K11
K12
K13
.26
.15
.22
.32
.36
.21
.51
.58
.58
.64
.62
.30
Knowledge
e21
e20
e19
e18
e17
e16
e15
e14
e13
A1
A2
A3
A4
A5
A6
A7
A8
A9
.80
-.17
.77
.84
.87
.88
.91
.82
.86
Attitude
e28
.16
.07
.28
e29
Practice
P1
P2
P3
P4
P5
P6
.30
.46
.67
.06
.23
-.07
e22
e23
e24
e25
e26
e27
