# Supplementary material for: Knowledge, Attitudes, and Reported Practices Regarding Prediabetes Among Hypertensive Patients With Abdominal Obesity: A Cross‐Sectional Structural Equation Modeling Analysis
Source: J Diabetes Res. 2026 Oct 10;2026:3315859. doi: 10.1155/jdr/3315859 (PMC13653257; doi:10.1155/jdr/3315859)
Supplement: Supplementary file 1 [file JDR-2026-3315859-s001.zip › Appendix.docx]

|  | Questionnaire ID: | | |
| --- | --- | --- | --- |
|  | Dear Participant,  We are researchers from Nantong Maternal and Child Health Care Hospital. We sincerely invite you to participate in our research study. This study aims to understand the knowledge, attitudes, and practices (KAP) of patients with hypertension and abdominal obesity regarding prediabetes. The findings will provide a basis for developing scientific intervention strategies, which may help improve the health outcomes of more people in the future. Your participation is voluntary. This study has been approved by the Ethics Review Committee. If you agree to participate, please note the following instructions:  1. Please complete the questionnaire. There are no right or wrong answers; simply respond based on your actual situation. Feel free to ask us any questions during the process. Please submit the questionnaire promptly upon completion.  2. This study involves a simple questionnaire survey and will not cause physical or psychological harm. However, it includes questions about personal information (e.g., gender, age). We will strictly maintain confidentiality and will not disclose your personal information. Please answer honestly.  3. As a participant, you have the right to access information related to this study and its progress. If you decide to withdraw at any time, please inform us. Your data will not be included in the research results.  Thank you sincerely for taking the time to support our scientific research!  □I have read the above information and agree to the use of my data for scientific research purposes.  Informed Consent Signature:  Date of Participation: Year ____ Month ____ Day ____ | | |
|  |  | | |
| **Part 1 Demographic Information** | | | |
| **1. Your gender** | | A. Male | B. Female |
| **2. Your age： .** | | | |
| **3. Your height：** **cm.** | | | |
| **4. Your weight： kg.** | | | |
| **5. Waist Circumference： cm.** | | | |
| **6. Blood Pressure Measurement：____ / ____mmHg (Systolic / Diastolic).** | | | |
| **7. Average Monthly Income:** | | A. Low income (< ¥2000)  B. Lower-middle income (¥2001 - ¥5000)  C. Upper-middle income (¥5001 - ¥8000)  D. High income (> ¥8000) | |
| **8. Education Level:** | | A. Primary school or below  B. Junior high school  C. Senior high school / Technical secondary school  D. Associate/Bachelor’s degree or above | |
| **9. Marital Status:** | | A. Unmarried  B. Married  C. Divorced  D. Widowed | |
| **10. Have you been diagnosed with hypertension and are currently taking medication?** | | A. Yes  B. Diagnosed with hypertension but not on medication  C. No | |
| **11. Have you been diagnosed with diabetes?** | | A. Yes  B. No  C. Not sure | |
| **12. Do you have a family history of diabetes?** | | A. Yes  B. No | |
| **13. Do you smoke?** | | A. Yes  B. No | |
| **14. Do you consume alcohol?** | | A. Yes  B. No | |

**Part 2 Prediabetes Knowledge Assessment**

**Please select the option that best reflects your understanding. Choose "Uncertain" if unsure of the answer.**

| 1. Are you aware of the concept of "prediabetes"? | A. Yes B. No |
| --- | --- |
| **True/False Questions:** | |
| 2. Prediabetes refers to blood sugar levels higher than normal but below the diagnostic threshold for diabetes. | A. True B. False C. Not sure |
| 3. Fasting blood glucose levels between 5.6-6.9 mmol/L fall within the normal range. | A. True B. False C. Not sure |
| 4. Prediabetes requires no intervention. | A. True B. False C. Not sure |
| 5. Individuals with prediabetes need regular blood glucose monitoring. | A. True B. False C. Not sure |
| 6. Prediabetes increases the risk of cardiovascular disease. | A. True B. False C. Not sure |
| 7. Hypertensive patients with abdominal obesity diagnosed with prediabetes should immediately start glucose-lowering medication. | A. True B. False C. Not sure |
| 8. Blood tests are typically recommended for detecting prediabetes. | A. True B. False C. Not sure |
| 9. Family history of diabetes, abdominal obesity, advanced age, and long-term high-sugar diets are associated with increased risk of prediabetes. | A. True B. False C. Not sure |
| 10. Regular physical activity, healthy diet, reduced salt intake, and periodic health check-ups can help hypertensive patients with abdominal obesity lower their risk of prediabetes. | A. True B. False C. Not sure |
| 11. Prediabetes increases the risk of cerebrovascular diseases and diabetes. | A. True B. False C. Not sure |
| 12. Adequate intake of high-fiber foods and fresh fruits/vegetables helps control blood sugar and weight, thereby aiding prediabetes management. | A. True B. False C. Not sure |
| 13. Prediabetes may present with non-specific symptoms like dry mouth, blurred vision, weight loss, or no symptoms at all. | A. True B. False C. Not sure |

| **Part 3 Attitudes Toward Prediabetes**  Please indicate your level of agreement with each statement by selecting one option from "Strongly Agree" to "Strongly Disagree". | | | | | | | | |
| --- | --- | --- | --- | --- | --- | --- | --- | --- |
| 1. I believe understanding prediabetes is crucial for preventing diabetes. | a. strongly agree | | | b. agree | c. neutral | d. disagree | e. strongly disagree | |
| 2. I think prediabetes does not require special attention. | a. strongly agree | | | b. agree | c. neutral | d. disagree | e. strongly disagree | |
| 3. I am willing to modify my lifestyle to prevent prediabetes. | a. strongly agree | | | b. agree | c. neutral | d. disagree | e. strongly disagree | |
| 4. I consider regular health check-ups important for early detection of prediabetes. | a. strongly agree | | | b. agree | c. neutral | d. disagree | e. strongly disagree | |
| 5. I believe individuals with prediabetes should seek guidance from healthcare professionals. | a. strongly agree | | | b. agree | c. neutral | d. disagree | e. strongly disagree | |
| 6. If diagnosed with prediabetes, I would actively take measures to control my blood sugar. | a. strongly agree | | | b. agree | c. neutral | d. disagree | e. strongly disagree | |
| 7. I think dietary modifications are essential for managing prediabetes. | a. strongly agree | | | b. agree | c. neutral | d. disagree | e. strongly disagree | |
| 8. I believe increased physical activity can effectively prevent prediabetes. | a. strongly agree | | | b. agree | c. neutral | d. disagree | e. strongly disagree | |
| 9. I would undergo regular prediabetes screening if recommended by a doctor. | a. strongly agree | | | b. agree | c. neutral | d. disagree | e. strongly disagree | |
| **Part 4 Behavioral Practices Related to Prediabetes**  Please indicate how frequently you engage in each behavior by selecting one option from "Never" to "Always". | | | | | | | |  |
| 1. How often do you engage in moderate-intensity exercise weekly? (e.g., brisk walking, swimming) | | a. never | | b. rarely | c. occasionally | d. frequently | e. always |  |
| 2. How often do you monitor your blood glucose weekly? | | a. never | | b. rarely | c. occasionally | d. frequently | e. always |  |
| 3. How often do you read educational materials about diabetes weekly? | | a. never | | b. rarely | c. occasionally | d. frequently | e. always |  |
| 4. How often do you consume alcoholic beverages weekly? | | a. never | | b. rarely | c. occasionally | d. frequently | e. always |  |
| 5. How often do you include whole grains and high-fiber foods in your weekly diet? | | | a. never | b. rarely | c. occasionally | d. frequently | e. always |  |
| 6. How often do you eat distractedly while watching TV/using your phone weekly? | | | a. never | b. rarely | c. occasionally | d. frequently | e. always |  |
